# HawkRank: a new scoring function for protein–protein docking based on weighted energy terms

**DOI:** 10.1186/s13321-017-0254-7

**Published:** 2017-12-28

**Authors:** Ting Feng, Fu Chen, Yu Kang, Huiyong Sun, Hui Liu, Dan Li, Feng Zhu, Tingjun Hou

**Affiliations:** 1 College of Pharmaceutical Sciences, Zhejiang University, Hangzhou, 310058 Zhejiang China; 2 State Key Lab of CAD&CG, Zhejiang University, Hangzhou, 310058 Zhejiang China

**Keywords:** Protein–protein interaction, Docking, Scoring, HawkRank

## Abstract

Deciphering the structural determinants of protein–protein interactions (PPIs) is essential to gain a deep understanding of many important biological functions in the living cells. Computational approaches for the structural modeling of PPIs, such as protein–protein docking, are quite needed to complement existing experimental techniques. The reliability of a protein–protein docking method is dependent on the ability of the scoring function to accurately distinguish the near-native binding structures from a huge number of decoys. In this study, we developed HawkRank, a novel scoring function designed for the sampling stage of protein–protein docking by summing the contributions from several energy terms, including van der Waals potentials, electrostatic potentials and desolvation potentials. First, based on the solvation free energies predicted by the Generalized Born model for ~ 800 proteins, a SASA (solvent accessible surface area)-based solvation model was developed, which can give the aqueous solvation free energies for proteins by summing the contributions of 21 atom types. Then, the van der Waals potentials and electrostatic potentials based on the Amber ff14SB force field were computed. Finally, the HawkRank scoring function was derived by determining the most optimal weights for five energy terms based on the training set. Here, MSR (modified success rate), a novel protein–protein scoring quality index, was used to assess the performance of HawkRank and three other popular protein–protein scoring functions, including ZRANK, FireDock and dDFIRE. The results show that HawkRank outperformed the other three scoring functions according to the total number of hits and MSR. HawkRank is available at http://cadd.zju.edu.cn/programs/hawkrank.

**Electronic supplementary material:**

The online version of this article (10.1186/s13321-017-0254-7) contains supplementary material, which is available to authorized users.

## Background

Protein–protein interactions (PPIs) are involved in a wide variety of biological processes, such as signal transduction [1, 2], transmembrane transport [3, 4], and antibody-antigen pairing [5, 6]. Deciphering structural and energetic determinants of PPIs is a prerequisite to understanding the PPIs-mediated functions in living cells. Unfortunately, only a tiny fraction of protein–protein complex structures have been characterized by high-resolution experimental techniques, such as X-ray crystallography, solution nuclear magnetic resonance (NMR) spectroscopy and cryo-electron microscopy (cryo-EM), which cannot keep pace with the growing demand in structure-based interactome analysis. Moreover, many weak and/or transient PPIs that play essential roles in regulating dynamic networks in bio-systems cannot be easily captured by experiments due to their unstable nature. On that account, computational approaches, especially protein–protein docking, are expected to provide an alternative and efficient way based on the unbound protein structures for predicting the binding complexes and understanding the recognition mechanisms at the atomic level [7–9].

The ultimate goal of protein–protein docking is the prediction of a near-native structure of the complex from many docking decoys, which generally falls into two stages: sampling and refinement. In the sampling stage, a large number of docking poses are generated and scored by various scoring functions; and in the refinement stage, the top-hit poses (or decoys) given by the first stage are re-scored and re-ranked by more rigorous scoring functions. Apparently, the success of protein–protein docking is, to a large degree, dependent on the ability of the scoring function to score and rank the decoys accurately. So far a large number of scoring functions have been developed, ranging from force field-based scoring functions such as ZRANK and FireDock [10–13], to knowledge-based ones such as dDFIRE and InterEvScore [14–16] and machine-learning scoring functions [17, 18]. However, recognizing near-native structures from a huge pool of alternatives is still quite challenging because the accuracy of most scoring functions needs to be improved. Besides, the ease of use, efficiency and general utility of the scoring functions should also be taken into account. Since the establishment of the Critical Assessment of PRedicted Interactions (CAPRI) campaign [19] in 2001 offers a community-wide platform that assesses the accuracy of protein–protein docking approaches, all related scoring functions and algorithms can be evaluated by comparing the submitted structures with the unpublished crystal structures from wide range of participants including predictors, servers and scorers. In 2010, Kastritis and Bonvin assessed the performances of 9 commonly used scoring functions and a free energy prediction algorithm on their ability to predict the binding affinities for 81 complexes [20]. They found that all the tested scoring functions could not provide reliable predictions because they all failed to correlate the experimental binding affinities (p*K*_*d*_) with the scores predicted by the corresponding scoring function, with the highest correlation of only − 0.32. Recently, our group analyzed the prediction results for the 24 targets tested from ROUND14 to ROUND 28 of CAPRI [21], and we found that, although the scorers perform better than the uploaders and predictors, they could give relatively high success rates (> 50%) for only two targets. Therefore, more approaches should be explored in order to improve the prediction accuracy of scoring functions for more reliable protein–protein docking.

In the past decade, more theoretically rigorous free energy calculation methods, such as Molecular Mechanics/Poisson Boltzmann Surface Area (MM/PBSA) and Molecular Mechanics/Generalized Born Surface Area (MM/GBSA), have been employed to predict binding affinities and identify correct binding structures for protein–protein systems [22–29]. For example, in our previous study [22], we evaluated the performances of MM/PBSA and MM/GBSA to predict the binding affinities and recognize the near-native binding structures for more than forty protein–protein complexes. The results show that, compared with most scoring functions used in protein–protein docking, MM/GBSA achieved better accuracy to predict the correct binding modes and binding affinities for the studied protein–protein systems. Therefore, the desolvation energy, which is related to the leading role of solvent exclusion during the protein inter-molecular assembly, is critical to identify these correct binding poses.

Although MM/GBSA is of more computational efficiency than other end-state free-energy calculation methods like thermodynamic integration (TI) and free energy perturbation (FEP), it is still much more time-consuming than the commonly used scoring functions in protein–protein docking, such as ZRANK, which treats the desolvation energy term with Atomic Contact Energy (ACE) model [30]. The computational cost in MM/GBSA is mainly attributed to the calculation of the polar desolvation energy term based on the GB model. In that regard, we developed HawkRank, a force field-based scoring function in which the energy terms are similar to those in MM/GBSA. Besides the frequently used van der Waals and electrostatic potentials, a simplified aqueous solvation model based on SASA (solvent accessible surface areas) was implemented into our scoring function. HawkRank is designed for the sampling stage of protein–protein docking and it can score a huge number of docked structures with low computational cost and high efficiency. We developed and benchmarked the present scoring function based on 176 high-resolution protein–protein complexes that are nonredundant at the family–family pair level. Compared with ZRANK, FireDock and dDFIRE, HawkRank performs consistently best on both the total number of hits and the (modified) success rate.

## Methods

HawkRank was developed by combining the weighted van der Waals potentials, electrostatic potentials and desolvation potentials. The workflow of the development of HawkRank is discussed below in details.

### Preparation of the protein–protein decoy dataset

More and more protein–protein complexes have been discovered, researchers classify protein–protein complexes based on various angles. The most common is that classify protein–protein complexes by protein family. The other researchers classify complexes as homo- and hetero-oligomeric complexes, non-obligate and obligate complexes and transient and permanent complexes, by the type of protein–protein interaction [31]. Besides, some researchers also excavate many effective statistical knowledge from the interface of the protein–protein interaction, such as the reported by Ref [32] and Ref [33]. Therefore, collecting a protein–protein complex database is a challenging task, by reason that, comprehensive consideration including protein family, the type of protein–protein interaction or characteristics of interface is need. However, there are still some databases pick out protein–protein complexes for theoretical research, such as protein–protein complexes in PDBbind [34], 2P2I-DB [35, 36], ZDOCK benchmark [37] and etc. The protein–protein complexes in ZDOCK benchmark 4.0 [38] were chose to develop the HawkRank scoring function in our study. ZDOCK benchmark 4.0 provides 176 nonredundant protein–protein complexes with high-resolution X-ray or NMR structures in bound and unbound states at the family–family pair level, including 124 complexes in the previous version 3.0 plus 52 newly-added ones. Besides, the protein–protein complex dataset used in training ZARNK and FireDock is the same ZDOCK benchmark series. More than that, the structured files of predictions docked by the unbound receptor and ligand from the ZDCOK benchmark are available from Zlab official website (https://zlab.umassmed.edu/zdock/benchmark.shtml), which is convenience for the training of scoring function. Therefore, we choose ZDOCK benchmark 4.0 for its convenience and the rationality of comparing HawkRank with ZRANK and FireDock. In this study, the 124 complexes were used as the training set to develop HawkRank and the other 52 ones as the test set to validate the actual performance of HawkRank. It should be noted that the benchmark 4.0 only contains binary interactions, so HawkRank is not suitable for the interactions between more than two proteins. Besides, HawkRank is also not suitable for the interactions between protein and peptide.

ZDOCK (version 3.0) was used to generate the decoys for each complex. ZDOCK systematically evaluates a huge number of docked conformations on a grid by using a combination of shape complementarity, electrostatics and statistical potential terms for scoring [39], and the search process is accelerated by the Fast Fourier Transformation (FFT) algorithm [11]. Depending on the sampling density in the rotational space (15° or 6°), ZDOCK can output 3600 or 54,000 predictions for each system. Benchmark 4.0 was downloaded from Zlab official website. For each system, the unbound RCSB Protein Data Bank (PDB) files of the receptor and ligand are provided in Benchmark 4.0. Cases in Benchmark 4.0 have been docked using ZDOCK3.0 and the results depending on the sampling density in the 6° rotational space are deposited in decoys_bm4_zd3.0_6 deg package file which can be download from Zlab official website. The missing hydrogen atoms in the unbound structures were added by using the *reduce* program (version 3.24) [40]. The decoys for each complex were generated by the Perl script in decoys_bm4_zd3.0_6 deg offered by ZDOCK, and a total of 54,000 decoys sorted by the ZDOCK scores were generated for each system. It should be noted that for each system only the top scored 10,000 decoys were used in our analysis.

### Criteria to evaluate the performance of protein–protein docking

Generally, in a protein–protein complex, the smaller protein is defined as the ligand protein and the larger one as the receptor protein. In our study, two types of root mean square deviations (RMSDs) between the predicted structure and the corresponding crystal structure, including ligand RMSD (L_RMSD) and interface RMSD (I_RMSD), were used as the criteria to evaluate the performance of protein–protein docking. L_RMSD, which is calculated over the C_*α*_ atoms of the ligand proteins when the receptors are superposed, was used to assess the global geometric fit between the predicted and native conformations [41]. I_RMSD, calculated over the C_*α*_ atoms of the interfacial residues when the predefined interfacial residues are superposed, was used to measure the geometric fit of the interface regions [41]. The interfacial residues in a protein–protein complex are defined as the residues within 10 Å of any atom in another protein [42]. The L_RMSDs and I_RMSDs were calculated by using the *ProFit* program [43], which employs the McLachlan algorithm in fitting. The structures of the protein–protein complexes were predicted from the unbound proteins, and therefore the structures of the proteins in the crystal complexes and the predicted complexes may have obvious difference.

The criteria to evaluate the performance of protein–protein docking for Target 107 in Round 35 of the CAPRI campaign are summarized in Additional file 1: Table S1. Based on these criteria, the predictions can be classified into several categories: incorrect, acceptable, medium, and high quality predictions. In this study, the hits are the predictions with L_RMSD less than 10 Å or I_RMSD less than 4 Å, which follows the criteria used in CAPRI.

### Parameterization of the SASA-based solvation model

**Fig. 1:**
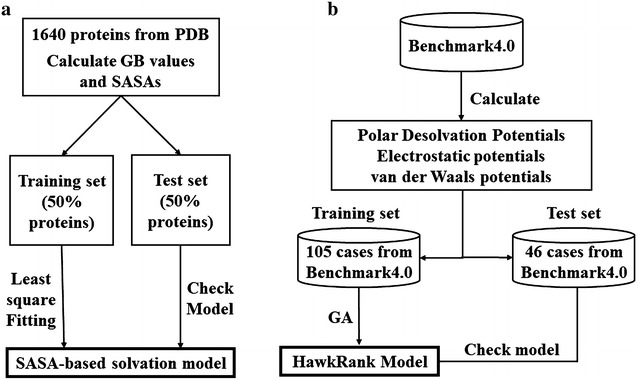
Workflow of the development of **a** the SASA-based solvation model and **b** the HawkRank scoring function

Because of the high computational cost of MM/GBSA, particularly the GB calculation, the use of MM/GBSA as a scoring function to rank thousands to even millions of docked conformations is computationally unaffordable. In order to balance computational cost and prediction accuracy, a novel SASA-based solvation model was developed by fitting the solvation free energies of proteins predicted by GB, as shown in Fig. 1a.

#### Generation of the protein dataset for the GB calculation

A protein dataset was established for the GB calculations (referred to as the GB dataset). The 1640 proteins in this dataset were selected from PDB based on the following criteria: (1). the structures are obtained by X-ray experiment, (2). the resolution should be lower than 2 Å, (3) the proteins do not contain any small molecule ligands, (4). the proteins are asymmetric, and (5) the proteins do not contain any modified residue.

Besides, for multiple structures whose sequences have at least 30% sequence identity, only a single structure is included in the dataset. Moreover, the protein structures with multiple conformations were eliminated.

#### Calculation of polar solvation free energies based on the GB model

The electrostatic/polar solvation free energies for the proteins in the GB dataset were computed by using the GB model implemented in Amber14. First, all missing heavy atoms and hydrogens of the proteins were added by using the *tleap* program in Amber14, and then the partial charges and force field parameters of the ff14SB force field were assigned. Subsequently, all proteins were optimized by 5000 cycles of minimization (2500 cycles of steepest descent and 2500 cycles of conjugate gradient). At last, the electrostatic solvation free energy for each protein was computed by using the modified GB model developed by Onufriev and colleagues (referred to as GB^*OBC1*^) [44]. A value of 80 was used for the exterior dielectric constant, and 1 was used for the solute dielectric constant.

#### Definition of atom types

Based on the study reported by Hou et al. [45], all atoms in the 20 standard amino acids were classified into 21 atom types shown in Table 1. The atoms that have similar chemical environment are defined as the same atom type.

**Table 1:**
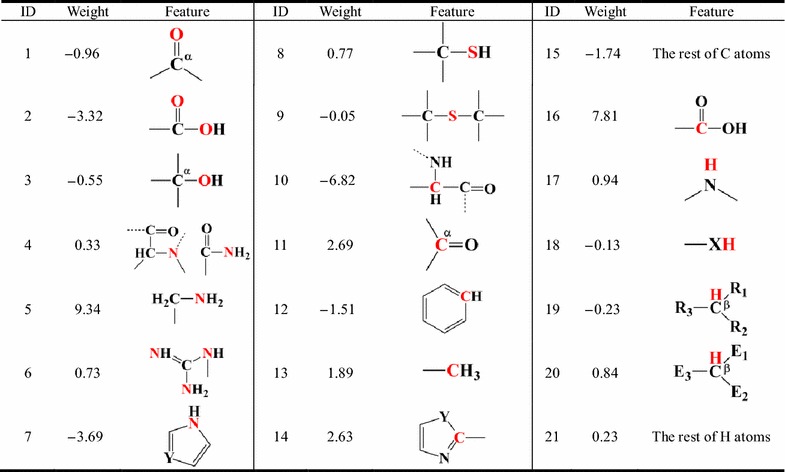
The definitions of the 21 atom types of proteins used in the SASA-based solvation model

(1) α represents C that is connected with only one O atom; (2) β represents C with sp3 hybridization; (3) X is O or S atom; (4) Y is N or C atom; (5) R is H or C atom; (6) Atoms at E1, E2 and E3 are N, O or S

#### Parameterization of the SASA-based solvation model

For each protein in the GB dataset, the SASAs were calculated by the NSC function that is an implementation of the DCLM (Double Cubic Lattice Method) variant of the Shrake’s and Rupley’s algorithm for surface calculation [46]. The van der Waals radii for H, C, N, O and S are listed in Additional file 1: Table S2 [47], and the radius of the solvent probe was defined to 0.6 Å, which performed best to develop the solvation model based on SASAs [48]. Consequently, the SASAs for the 21 different atom types could be calculated, and the SASA-based solvation model given by Equation function (1) was developed.

1 $$psol_{SASA} = w_{0} N_{res} + \sum\limits_{i = 1}^{21} {w_{i} SASA_{i} + {\text{constant}}} \quad i = \text{ }1,\text{ }2,\text{ }3 \ldots 21$$

where *psol*_*SASA*_ represents the polar component of solvation free energy; *i* is the IDs (Table 1) of the atom types for a given protein; *w*_*i*_ is the weight for atom type *i*; *SASA*_*i*_ is the sum of SASAs for atom type *i* in the given protein; *N*_*res*_ represents the total number of the residues in a given protein, and *w*_*0*_ is the weight of *N*_*res*_; constant is the constant term in this formula. The whole GB dataset was randomly split into a training set (~ 800 protiens) with 50% of the proteins and a test set with the remaining proteins. Least-square fitting was applied to optimize the weights of different atom types and *N*_*res*_ based on the training set, and then the reliability of the developed solvation model was evaluated by the consistency between the predictions given by the GB model and those given by the SASA-based model to the test set.

### Development of the HawkRank scoring function

The HawkRank scoring function is a linear weighted sum of the van der Waals attractive and repulsive potentials, the electrostatic attractive and repulsive potentials, and the desolvation potentials. The core formula of HawkRank is shown in Equation function (2), where Δ*E* is the value of potentials and *w* is the weight of corresponding potentials:

2 $$\begin{aligned} {\text{HawkRank Score}} & = w_{{vdW_{attr} }}\Delta E_{{vdW_{attr} }} + w_{{vdW_{repu} }}\Delta E_{{vdW_{repu} }} \\ & \quad + w_{{elec_{attr} }}\Delta E_{{elec_{attr} }} + w_{{elec_{repu} }}\Delta E_{{elec_{repu} }} \\ & \quad + w_{pdsol}\Delta E_{pdsol} \\ \end{aligned}$$

The details about the energy terms used in the scoring function are described below. The workflow of the development of HawkRank is shown in Fig. 1b.

#### Calculation of the van der Waals potentials

The definition of the van der Waals potentials (Equation function 3) is the same to that used in Kortemme’s study [49]. The formula was modified from the classical 6-12 Lennard-Jones potential. As shown in Equation function (3), the van der Waals potentials are split into the attractive and repulsive parts. The repulsive part was calculated by the linear formula to reduce the potential local clashes caused by the discrete rotamers of side chains and fixed backbones [49], and the attractive part was defined as the traditional formula of the classical 6–12 Lennard-Jones potential. To improve computational efficiency, only the van der Waals interactions between two atoms with distance less than 12 Å were calculated.

3 $$\begin{aligned} &E_{attr} = \varepsilon_{ij} \left\{ {\left( {\frac{{\sigma_{ij} }}{{r_{ij} }}} \right)^{12} - 2\left( {\frac{{\sigma_{ij} }}{{r_{ij} }}} \right)^{6} } \right\}\quad \text{for}\,0.89\sigma_{ij} < r_{ij} < 12\;{\text{\AA}} \hfill \\ & E_{repu} = 10.0\left( {1 - \frac{{r_{ij} }}{{0.89\sigma_{ij} }}} \right)\quad \text{for}\,0.89\sigma_{ij} > r_{ij} \hfill \\ \end{aligned}$$

where *i* and *j* are atoms *i* and *j*, *r*_*ij*_ is the interatomic distance between them, *σ*_*ij*_ is the sum of atomic radii, and *ε*_*ij*_ is the well depth derived from the Amber ff14SB force field [50].

#### Calculation of the electrostatic potentials

For the electrostatic potentials, the standard Coulomb equationis used (Equation function 4):

4 $$E_{elec} = 332\frac{{q_{i} q_{j} }}{{\varepsilon r_{ij} }}$$

where *i* and *j* are atoms *i* and *j*, *r*_*ij*_ is the interatomic distance between them, *q*_*i*_ and *q*_*j*_ are the atomic partial charges afforded by the Amber ff14SB force field [50]. The dielectric constant *ε* was set to 1. The electrostatic potentials are also split into an attractive and a repulsive parts. If the *E*_*ele*_ between two atoms is less than zero, it will be added into the electrostatic attractive potentials, and otherwise added into the electrostatic repulsive potentials.

#### Calculation of desolvation potentials

The SASA-based solvation model developed in this study was used to compute the solvation potentials of the unbound receptor, unbound ligand and complex for each system. Then, the desolvation (Δ*E*_*pdsol*_) was calculated by taking the polar solvation free energy of complex (*psol*_*SASA(com)*_) to subtract the sum of the polar solvation free energies of receptor (*psol*_*SASA(rec)*_) and ligand (*psol*_*SASA(lig)*_) (Equation function 5).

5 $$\Delta E_{pdsol} = psol_{{SASA\left( {com} \right)}} - \left( {psol_{{SASA\left( {lig} \right)}} + psol_{{SASA\left( {rec} \right)}} } \right)$$

#### Training the scoring function

For each case in ZDOCK benchmark 4.0, the I_RMSDs and L_RMSDs for the top 10,000 decoys were calculated. After eliminating those cases whose hits could not be found in the top 10,000 decoys, 151 cases out of 176 were included in the whole dataset. The 105 cases out of the 124 found in ZDOCK benchmark 3.0 were put into the training set and the remaining 46 cases into the test set. Then, based on the training set, genetic algorithm (GA) implemented in the R package (genalg) was used to determine the optimal set of the weights for the energy terms used in HawkRank. We set the maximum (3.0) and minimum (0.0) value for the weight of the van der Waals attractive potentials, electrostatic attractive potentials, electrostatic repulsive potentials and the polar desolvation potentials. However, we set the maximum (0.001) and minimum (0.0) value for the weight of the van der Waals repulsive potentials, on account of inherent defect in the equation function to calculate the van der Waals repulsive potentials and we want to reduce the impact of the van der Waals repulsive potentials on the whole scoring function. Besides, the population size was set as 200, the number of iterations was set as 1500 and the number of chromosomes that are kept into the next generation was about 20% of the population size.

### Evaluation of the performance of HawkRank

The capability of HawkRank to recognize the near-native poses from the decoys was compared with those of two popular force field-based scoring functions used in protein–protein docking, ZRANK [11] and FireDock [12], and a knowledge-based scoring function named dDFIRE [15].

ZRANK is a force field-based scoring function that is a linear combination of atom-based potentials, including electrostatics, van der Waals, and desolvation potentials. Pairwise Atomic Contact Energy (ACE) model [30] is used to calculate the desolvation energy. Parameters used to calculate the van der Waals and desolvation potentials in the ZRANK scoring function are derived from the CHARMM 19 polar hydrogen force field.

FireDock is a method for the refinement and rescoring of rigid-body docking solutions. The function of FireDock includes ACE, softened van der Waals interactions, electrostatic interactions and internal energy. Moreover, hydrogen and disulfide bonds, π-stacking and aliphatic interactions are also considered [12]. Compared with ZRANK and HawkRank, FireDock includes more energy terms.

dDFIRE is an all-atom statistical and knowledge-based energy function. Each polar atom is treated as a dipole with a direction. The orientation of the dipole is defined by the bond vectors that connect the polar atoms with other heavy atoms, and the function of dDFIRE is extracted from protein structures based on the distance between two atoms and the three angles involved in dipole–dipole interactions [15]. Besides, the hydrogen bonding interactions are considered in dDFIRE via the physical dipole–dipole interactions.

It is well known that how to quantitatively evaluate the performance of a scoring function is quite essential. Traditionally, success rate (SR), the proportion of total cases with at least one hit in the top *N* predictions, has been widely used to evaluate the performance of a given scoring function [16]. However, SR has its own intrinsic deficiency. For example, for two different protein–protein complexes, one has nine hits in the top 100 predictions while the other has only one hit in the top 100 predictions. However, when we calculate SR for these two systems, the contributions of the predictions for these two complexes to SR are identical, but nonetheless, the capacity of the scoring function to correctly rank the predictions for these two complexes is quite different. Moreover, SR ignores the rank for each hit. The “top-ranking” method is also a popular way to evaluate the performance of a given scoring function by identifying the first hit in the ranked predictions and comparing their ranks. The two evaluation strategies mentioned above are relatively intuitional, but not quite reasonable.

In order to evaluate the ranking performance of a scoring function on the whole benchmark in a reasonable way, MSR (modified success rate) proposed by us (Equation function 6) [21] was used in this study.

6 $$Y = \sum {F(x_{i} )} /size(X)$$

7 $$F = \frac{{\sum\nolimits_{j = 1}^{N} {(1 + top_{j} )} }}{HIT}$$

8 $$top_{j} = \frac{1}{{rank_{j} }}$$

where *x*_*i*_ represents a protein–protein complex, size(*X*) is the total number of the studied systems in a benchmark, *HIT* equals to the total number of the hits for a protein–protein complex, *rank*_*i*_ is the rank number of a hit, *top*_*j*_ is the reciprocal of *rank*_*j*_, *Y* is the integrated value to evaluate the performance of the scoring function for multiple protein–protein complexes, and *F* is the value to evaluate the performance of the scoring function for a specific protein–protein complex.

## Results and discussion

### Performance of the SASA-based solvation model

**Fig. 2:**
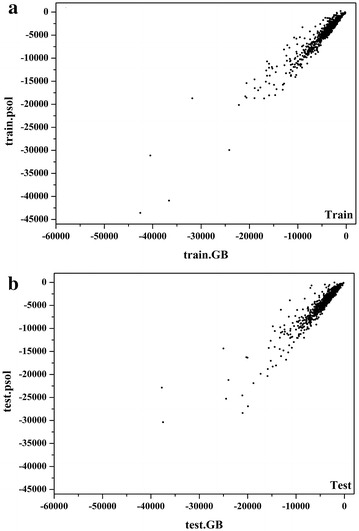
Comparison of the solvation free energies predicted by the GB model and the SASA-based solvation model for the **a** training set and **b** test set

On the whole, the SASA-based solvation model (Equation function 2) yields satisfactory results for the training set (*r* = 0.96). The plot of the polar solvation free energies predicted by the SASA-based model *versus* those predicted by the GB model is shown in Fig. 2a. Moreover, the model illustrates good predictive ability on the test set (*r* = 0.93, Fig. 2b), suggesting that the developed model is reliable and not overfitting. The weights (*w*_*i*_) for the 21 atom types derived by fitting the GB polar solvation free energies for the training set are listed in Table 1. The *w*_*0*_ and the constant term determined by fitting are − 10.51 and 59.78, respectively.

### Performance of HawkRank

**Fig. 3:**
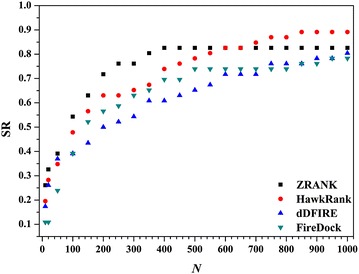
The Success Rate (SR) as a function of the number of the top predictions (*N*) for HawkRank, ZRANK, dDFIRE and FireDock

Based on the training set, the optimal weights for the five types of potentials were determined by GA as follows: 0.850 for the van der Waals attractive potentials, 0.0005 for the van der Waals repulsive potentials, 1.887 for the electrostatic attractive potentials, 1.853 for the electrostatic repulsive potentials, and 0.9303 for the polar desolvation potentials. Then, the trained HawkRank scoring function was used to score the decoys for the test set. Among the 46 cases in the test set, the correct binding structures for 2 cases (1CLV, 2AYO) are the best hits, those for 6 cases (1CLV, 1SYX, 2AYO, 2OUL, 3D5S, 4CPA) can be found in the top 5 ranked predictions and those for 13 cases can be found in the top 20 ranked predictions. Then, we calculated the SR values (*N* = 10, 20, 50, 100, 150, 200, 250, 300, 350, 400, 450, 500, 550, 600, 650, 700, 750, 800, 850, 900, 950, and 1000) for the 46 cases in the test set. As shown in Fig. 3, HawkRank’s SR is higher than 50% when *N* is larger than 150 while ZRANK’s SR is higher than 50% when *N* is larger than 100. When *N* is larger than 600, HawkRank’s SR value equals to or exceeds ZRANK’s SR value. According to SR, the performances of HawkRank and ZRANK are comparative. However, HawkRank’s SR values are always higher than those of dDFIRE and FireDock.

Next, we analyzed the performance of each individual energy term used in HawkRank from the view of MSR. The *Y* values of the five energy terms are shown in Additional file 1: Figure S1. The performances of the two repulsive potentials are not satisfactory, but the attractive ones perform much better, especially the electrostatic attractive potentials. When *N* ≤ 200, the polar desolvation potentials show good performance, and its performance decreases gradually with the increase of *N*.

**Fig. 4:**
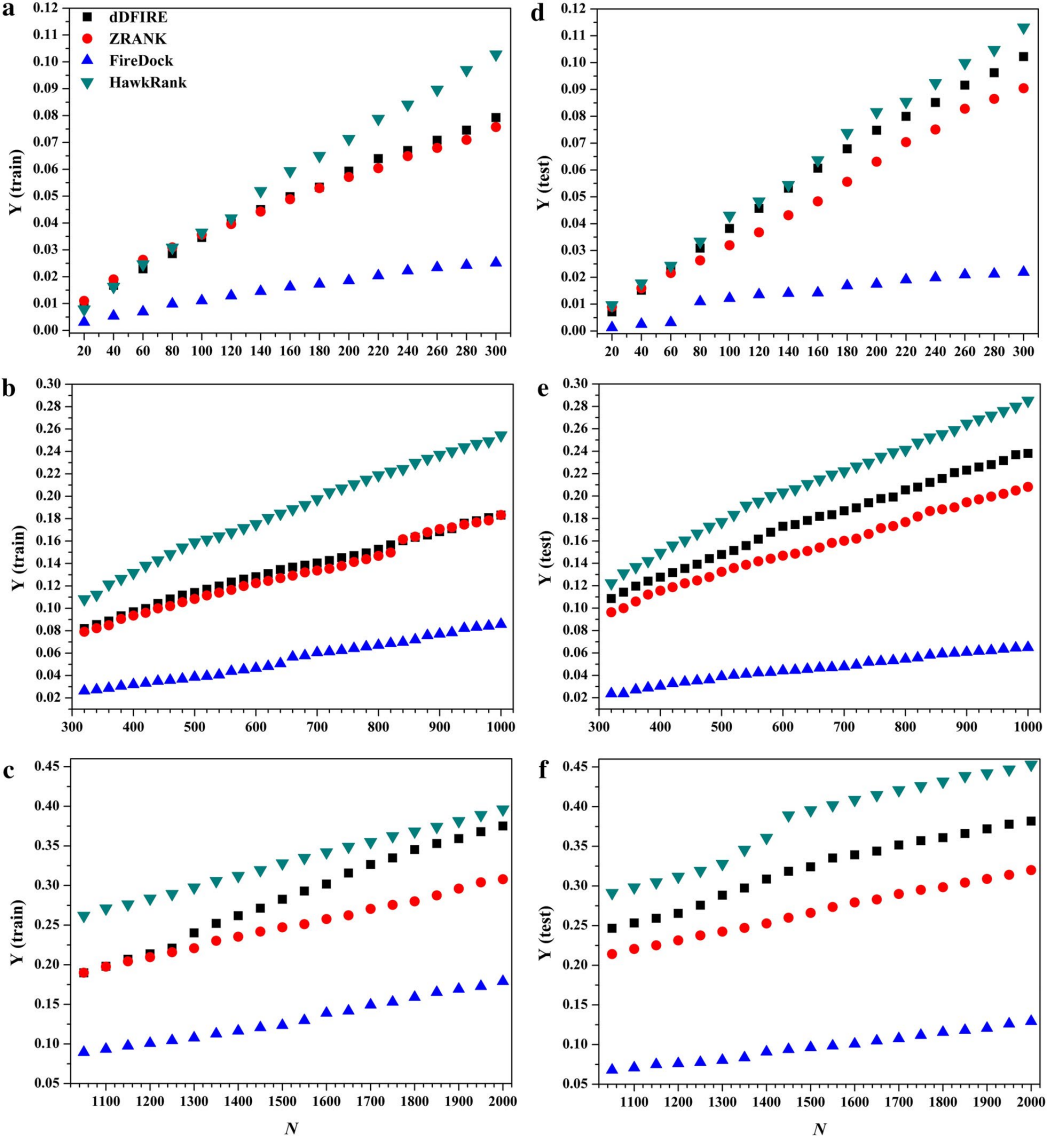
The *Y* curves for the four scoring functions: **a** *N* from 20 to 300 for the training set, **b** *N* from 20 to 300 for the test set, **c** *N* from 320 to 1000 for the training set, **d** *N* from 320 to 1000 for the test set, **e** *N* from 1050 to 2000 for the training set, and **f** *N* from 1050 to 2000 for the test set

The performance of HawkRank was then compared with those of ZRANK, FireDock and dDFIRE. The *Y* values (from *N* = 20 to *N* = 2000) were calculated for each scoring function. Overall, as shown in Fig. 4, HawkRank outperforms ZRANK, FireDock and dDFIRE for both of the training set and test set. In details, when *N* is small than 160, HawkRank shows comparative performance with ZRANK and dDFIRE, but when *N* becomes higher than 160 it performs better. With *N* increases, the *Y* values of HawkRank increase much faster than those of the other three scoring functions. Therefore the HawkRank scoring function can identify more hits with better ranking in the top *N* = 160–20,000 than the other three scoring functions. Besides, the performance of Firedock performs apparently worse than the other three scoring functions (Fig. 4). In summary, we can make the following conclusion: for a force field-based scoring function, inclusion of more energy terms may not be necessary to achieve better ranking capabilities. The energy terms used in HawkRank are similar to those used in ZRANK while the major difference lies in the desolvation term. In HawkRank, a SASA-based solvation model was used to calculate the polar desolvation potentials, instead of the frequently-used ACE model used in ZRANK. The better performance of HawkRank over ZRANK was believed to be attributed to the better estimation of protein–protein desolvation free energy. Therefore we believe that, by fitting the solvation free energies predicted by GB, the SASA-based model should provide more reliable predictions than ACE.

### Comparison of Pearson correlations between RMSDs and scores

**Fig. 5:**
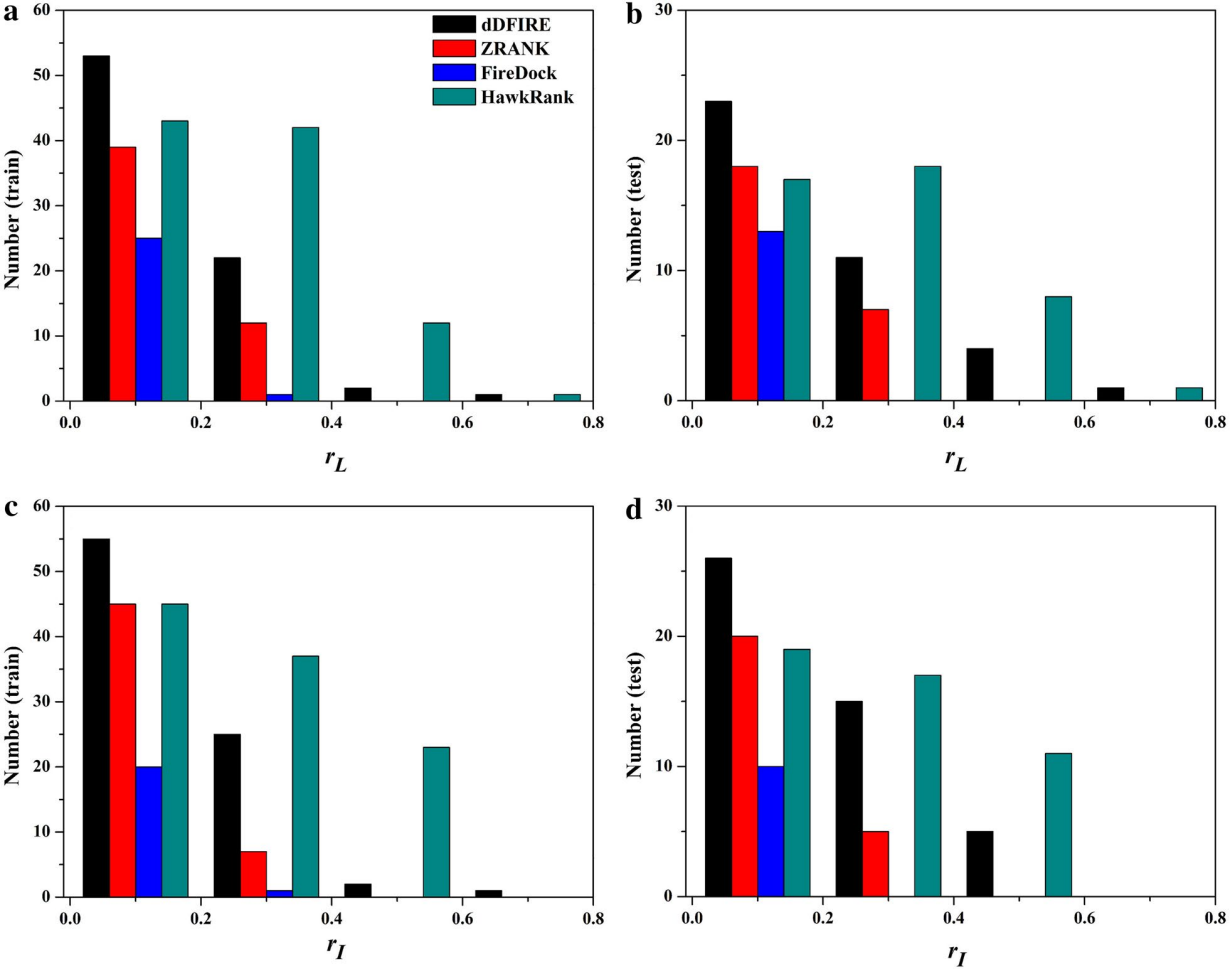
Number of the cases for each scoring function in four intervals: **a** number of cases about r_*L*_ for the training set, **b** number of cases about r_*L*_ for the test set, **c** number of cases about r_*I*_ for the training set, **d** number of cases about r_*I*_ for the test set

Generally, the smaller the RMSD between the predicted complex structure and its native conformation is, the more similar these two structures are. The calculation of RMSD requires a prior superimposition of the predicted complex structure onto its native conformation, but the superimposition that minimizes the global RMSD of the predicted complex to the native conformation may be not necessarily the best solution: such superimposition is often compromised by a small number of significantly deviating fragments [51]. Therefore, we cannot exclude the possibility that a good docked structure with low energy has relatively larger I_RMSD or L_RMSD. It is expected that a lower RMSD is associated with a better docking score and vice versa. Therefore for a good scoring function which has lower scores for better predictions, a positive correlation between RMSDs and the docking scores should be observed. Then, the correlations between I/L_RMSDs and the docking scores predicted by ZRANK, FireDock, dDFIRE or HawkRank were analyzed for the 176 cases in the dataset. Here, *r*_*I*_ is referred to as the correlation between the scores and I_RMSDs and *r*_*L*_ as the correlation between the scores and L_RMSDs. Here, the *r*_*I*_ or *r*_*L*_ values were partitioned into the following four intervals: 0 < *r*_*I*_/*r*_*L*_ ≤ 0.2, 0.2 < *r*_*I*_/*r*_*L*_ ≤ 0.4, 0.4 < *r*_*I*_/*r*_*L*_ ≤ 0.6, and 0.6 < *r*_*I*_/*r*_*L*_ ≤ 0.8. The results are shown in Fig. 5. Moreover, the cumulative occurrences of the *r*_*I*_/*r*_*L*_ values are listed Table 2. For HawkRank, *r*_*L*_ achieves 0.4 for 13 cases and *r*_*I*_ achieves 0.4 for 23 cases in the training set, and *r*_*L*_ achieves 0.4 for 9 cases and *r*_*I*_ achieves 0.4 for 11 cases in the test set. For dDFIRE, *r*_*L*_ achieves 0.4 for 3 cases and *r*_*I*_ achieves 0.4 for 3 cases in the training set, and *r*_*L*_ achieves 0.4 for 5 cases and *r*_*I*_ achieves 0.4 for 5 cases in the test set. ZRANK’s *r*_*I*_/*r*_*L*_ and Firedock’s *r*_*I*_/*r*_*L*_ even cannot achieve 0.4 for any case. Apparently, according to the cumulative occurrences of *r*_*I*_/*r*_*L*_ shown in Table 2, HawkRank achieves much better performance than the other three scoring functions.

**Table 2:** Number of the cases for each scoring function in different intervals of *r* ^a^

	dDFIRE		ZRANK		FireDock		HawkRank	
*Training set (124 cases)*								
0.6 < *r* ≤ 0.8	1^a^	1^a^	0	0	0	0	1	0
0.4 < *r* ≤ 0.8	3	3	0	0	0	0	13	23
0.2 < *r* ≤ 0.8	25	28	12	7	1	1	55	60
0.0 < *r* ≤ 0.8	78	83	51	52	26	21	98	105
*Test set (52 cases)*								
0.6 < *r* ≤ 0.8	1	0	0	0	0	0	1	0
0.4 < *r* ≤ 0.8	5	5	0	0	0	0	9	11
0.2 < *r* ≤ 0.8	16	20	7	5	0	0	27	28
0.0 < *r* ≤ 0.8	39	46	25	25	13	10	44	47

^a^For each scoring function, the first column is the number for *r*_*L*_, and the second column is the number for *r*_*I*_

**Fig. 6:**
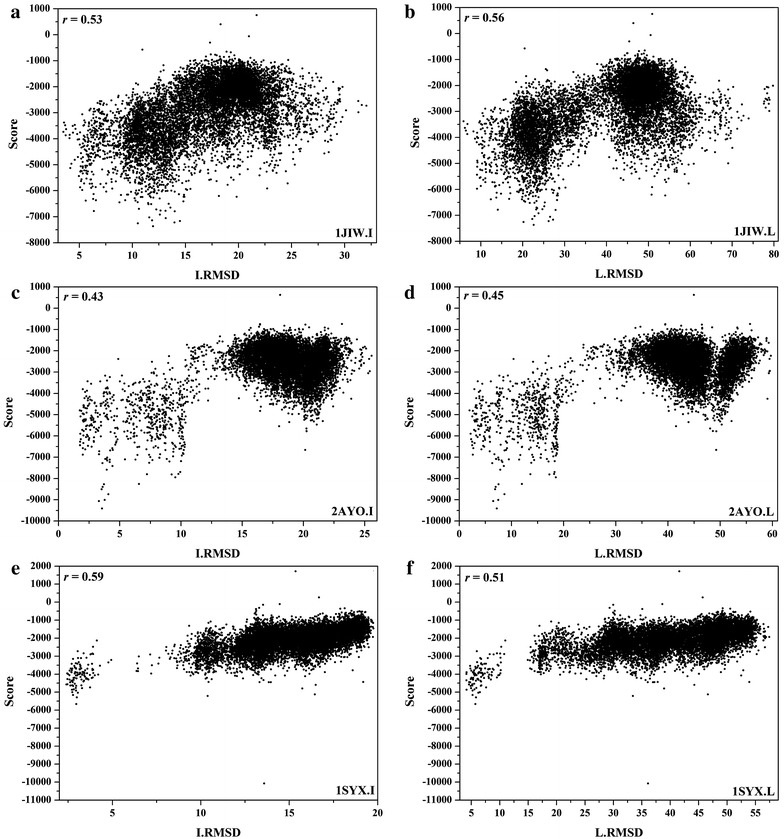
Correlations between I/L_RMSDs and the scores predicted by HawkRank for 1JIW, 2AYO and 1SYX (r_I_/r_L_ > 0.4): **a** the correlation between I_RMSDs and scores for 1JIW, **b** the correlation between L_RMSDs and scores for 1JIW, **c** the correlation between I_RMSDs and scores for 2AYO, **d** the correlation between L_RMSDs and scores for 2AYO, **e** the correlation between I_RMSDs and scores for 1SYX, **f** the correlation between L_RMSDs and scores for 1SYX

**Fig. 7:**
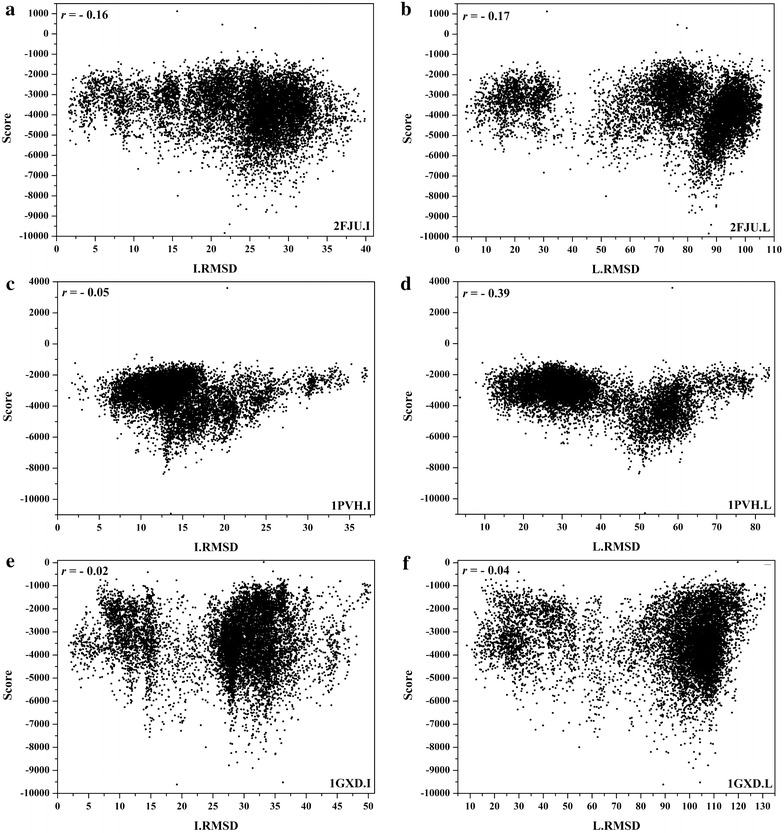
Correlations between I/L_RMSDs and the scores predicted by HawkRank for 2FJU, 1PVH and 1GXD (*r*_*I*_/*r*_*L*_ < 0): **a** the correlation between I_RMSDs and scores for 2FJU, **b** the correlation between L_RMSDs and scores for 2FJU, **c** the correlation between I_RMSDs and scores for 1PVH, **d** the correlation between L_RMSDs and scores for 1PVH, **e** the correlation between I_RMSDs and scores for 1GXD, **f** the correlation between L_RMSDs and scores for 1GXD

**Fig. 8:**
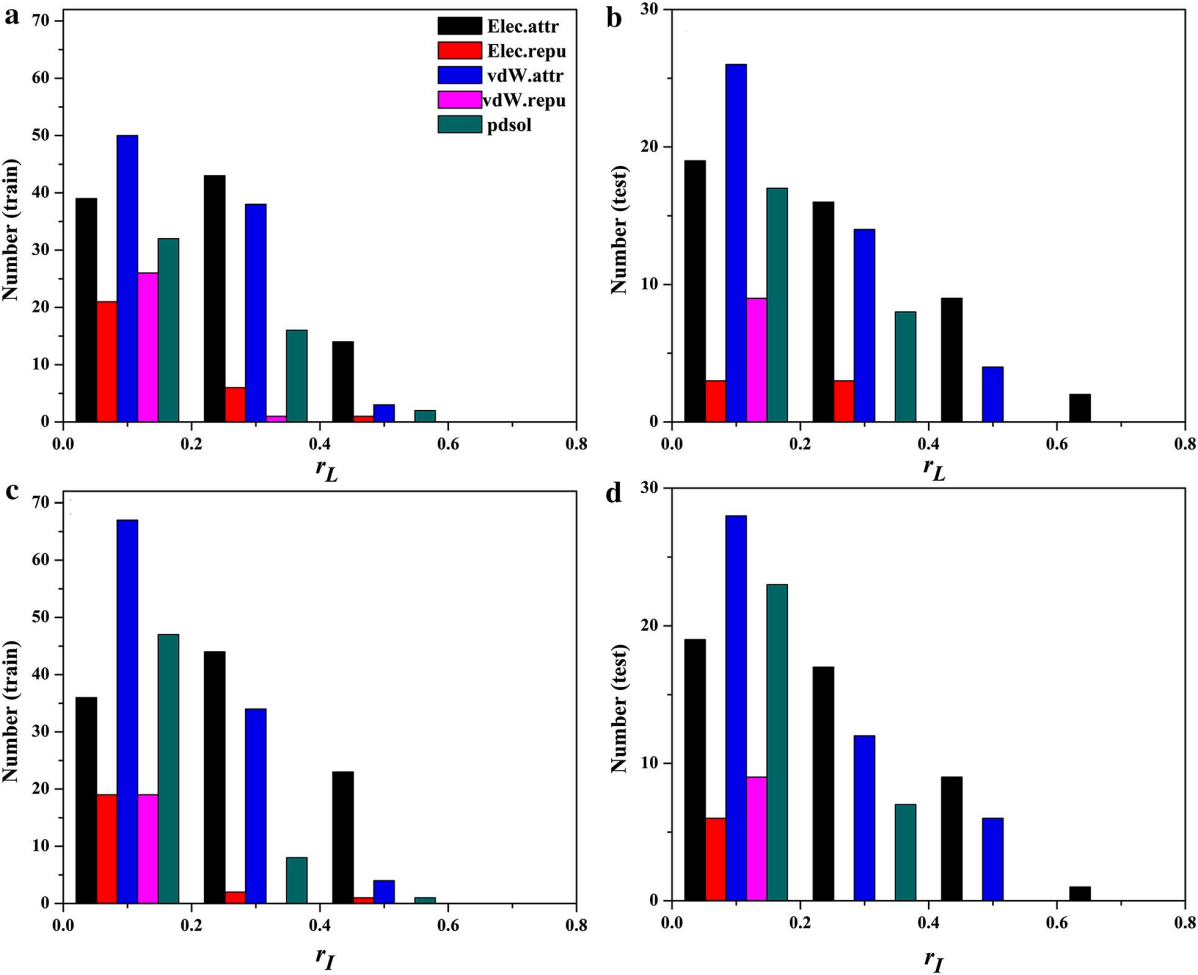
Number of the cases for each energy term of HawkRank in four intervals: **a** number of cases about r_*L*_ for the training set, **b **number of cases about r_*L*_ for the test set, **c** number of cases about r_*I*_ for the training set, **d** number of cases about r_*I*_ for the test set

Three examples (1JIW, 2AYO, and 1SYX) with rI/rL > 0.4 and three examples (2FJU, 1PVH, and 1GXD) with *r*_*I*_/*r*_*L*_ < 0 are shown in Figs. 6 and 7, respectively. In next section, we also found that 2AYO and 1SYX were predicted accurately while 2FJU and 1GXD were predicted inaccurately by HawkRank. We also calculated the correlations (*r*) between I/L_RMSDs and each energy term given by HawkRank, and the numbers of the cases in four intervals of *r* are also displayed in Fig. 8. It seems that the electrostatic attractive potentials have better correlation with I/L_RMSDs than the other energy terms, indicating that the electrostatic attractive potentials contribute more to the total scoring than any other energy term. In summary, the HawkRank scoring function provides a better funnel-shaped energy landscape than the other three scoring functions.

### Limitations of HawkRank

Although HawkRank has better ranking capability than ZRANK, FireDock and dDFIRE, it still has some limitations. The *F* (*N* = 500) values for the 46 tested cases with the hits found in the top 10,000 decoys are listed in Additional file 1: Table S3. The 46 cases are ranked by the HawkRank’s *F* values in ascending order. *F* = 0 means that the scoring function does not find any hit in the top 500 predictions. For dDFIRE, ZRANK, FireDock and HawkRank, the numbers of the cases with *F* = 0 are 16, 8, 12 and 10, respectively. For 1XU1, 1ZHH, 2B4 J and 2OOR, the four scoring functions cannot find any hit in their top 500 predictions. For 1GXD, 1JZD, 1US7 and 2FJU, more than 60 hits docked by ZDOCK, but HawkRank performs poorly on them.

The COCOMAPS online service [52] was used to analyze the protein–protein interfaces for the 46 cases in the test set. We found that the four cases (1GXD, 1JZD, 1US7 and 2FJU) that cannot be successfully predicted by HawkRank have quite low percentages of near-native interface areas relative to the total surface areas. The four cases (1SYX, 2AYO, 2OZA and 3D5S) that can be well predicted by HawkRank have relatively high percentages of near-native interface areas relative to the total surface areas. In other words, for the cases with the higher percentages of near-native interface areas, HawkRank may provide better ranking capability. Then, we used the COCOMAPS online service to analyze the interface characteristics of the best solutions predicted by HawkRank for 1GXD, 1JZD, 1US7 and 2FJU. As shown in Table 3, the percentages of the interface areas in the native complex structures for the four cases are much lower than those in the top ranked structures. Generally, for a protein–protein complex, larger interface area implies that the receptor and ligand can form more favorable surface complementary. Apparently, HawkRank is apt to recognize the decoys with larger interface area percentages in near-native structures. This is also the reason why HawkRank can find more hits in its top 500 predictions for these cases with larger native interface areas such as 1SYX, 2AYO, 2OZA and 3D5S.

**Table 3:** Comparative analysis of the interface areas for 1GXD, 1JZD, 1US7 and 2FJU

	Native percentage (%)^a^	Spurious percentage (%)^b^
1GXD	2.885	5.665
1JZD	3.650	9.005
1US7	2.405	8.765
2FJU	1.611	4.470

^a^The percentage of the near-native interface areas relative to the total surface areas of the native complex

^b^The percentage of the interface areas relative to total surface areas of the best solutions predicted by HawkRank

### Computational cost of HawkRank

The arithmetic speed of HawkRank is closely related to the size of the protein–protein complex. The speed of HawkRank is fast enough to meet the requirements for scoring a large number of decoys in the sampling stage of protein–protein docking. The scoring of a complex with 500 residues needs less than 0.3 s on a core (Intel Xeon CPU E5-2692 v2 @2.20 GHz) with Linux operating system.

## Conclusions

In this study, we developed a new scoring function named HawkRank by combining polar desolvation potentials, van der Waal potentials and electrostatic potentials. HawkRank introduces a fast and effective way to calculate the desolvation potentials based on a SASA-based solvation model. Compared with ZRANK, FireDock and dDFIRE, HawkRank shows better ranking capabilities to the 46 cases in test set. Besides, the scores predicted by HawkRank have higher correlations with L/I_RMSDs than those predicted by ZRANK, FireDock and dDFIRE, suggesting that the HawkRank scoring gives a better funnel-shaped energy landscape than the other three scoring functions. Although its prediction accuracy still needs to be improved for some protein–protein complexes with small interface areas, HawkRank is efficient to meet current requirements for scoring a large number of decoys in the sampling stage of protein–protein docking. In the light of the above assessment and the conclusion in our previous study that MM/GBSA rescoring has good capability to distinguish the correct protein–protein binding structures from the decoys, it would suggest to be an efficient protocol of using HawkRank followed by the MM/GBSA rescoring to improve the predictions of protein–protein docking.

## Additional file

**Additional file 1**: Contains the supplementary information of the manuscript. This includes **Table S1.** Criteria of assessing predictions in CAPRI (Å); **Table S2**. The van der Waals radii for H, C, N, O and S (Å); **Table S3**. The *F* values for the 46 cases in test set; **Figure S1.** The Y curves for the five energy terms in HawkRank: (A) *N* from 20 to 300 for the training set, (B) *N* from 20 to 300 for the test set, (C) *N* from 320 to 1000 for the training set, (D) *N* from 320 to 1000 for the test set, (E) *N* from 1050 to 2000 for the training set, and (F) *N* from 1050 to 2000 for the test set.
