# Supplementary material for: HawkRank: a new scoring function for protein–protein docking based on weighted energy terms
Source: J Cheminform. 2017 Dec 28;9:66. doi: 10.1186/s13321-017-0254-7 (PMC5745212; doi:10.1186/s13321-017-0254-7)
Supplement: Supplementary file 1 — Additional file 1: Contains the supplementary information of the manuscript. This includes Table S1. Criteria of assessing predictions in CAPRI (Å); Table S2. The van der Waals radii for H, C, N, O and S (Å); Table S3. The F values for the 46 cases in test set; Figure S1. The Y curves for the five energy terms in HawkRank: (A) N from 20 to 300 for the training set, (B) N from 20 to 300 for the test set, (C) N from 320 to 1000 for the training set, (D) N from 320 to 1000 for the test set, (E) N from 1050 to 2000 for the training set, and (F) N from 1050 to 2000 for the test set. [file 13321_2017_254_MOESM1_ESM.docx]

**Supplementary Materials**

**Table S1.**Criteria of assessing predictions in CAPRI (Å)

| Categories | Criterion1 | Criterion2 |
| --- | --- | --- |
| Incorrect prediction | f(nat)*^a^* < 0.1 | (L_RMSD > 10.0) and (I_RMSD > 4.0) |
| Acceptable prediction | ((f(nat) >= 0.1) and (f(nat) < 0.3)) and ((L_RMSD <= 10.0) or (I_RMSD <= 4.0)) | (f(nat) >= 0.3)and (L_RMSD > 5.0) and (I_RMSD > 2.0) |
| Medium prediction | ((f(nat) >= 0.3) and (f(nat) < 0.5)) and ((L_RMSD <= 5.0) or (I_RMSD <= 2.0)) | (f(nat) >= 0.5) and (L_RMSD > 1.0) and (I_RMSD > 1.0) |
| High quality prediction | (f(nat) >= 0.5) and ((L_RMSD <= 1.0) or (I_RMSD <= 1.0)) | None |

*^a^* f(nat) is the fraction of the ligand-receptor contacts of predictions that are also found in the native structure (both Rule 1 and Rule 2 can be used to determine the category).

**Table S2**. The van der Waals radii for H, C, N, O and S (Å)

| Element | Radius (Å) |
| --- | --- |
| Hydrogen | 1.2 |
| Carbon | 1.7 |
| Nitrogen | 1.55 |
| Oxygen | 1.52 |
| Sulfur | 1.8 |

**Table S3**. The *F* values for the 46 cases in test set

|  | Percentage (%)*^a^* | TotalHits*^b^* | *F* | | | | |
| --- | --- | --- | --- | --- | --- | --- | --- |
|  |  |  | dDFIRE | ZRANK | FireDock | HawkRank |  |
| 1GXD*^a^* | 2.885 | 66 | 0.673 | 0.490 | 0.030 | 0.000 |  |
| 1JZD*^a^* | 3.650 | 127 | 0.008 | 0.071 | 0.032 | 0.000 |  |
| 2FJU*^a^* | 1.610 | 138 | 0.000 | 0.007 | 0.022 | 0.000 |  |
| 1US7*^a^* | 2.405 | 68 | 0.000 | 0.074 | 0.044 | 0.000 |  |
| 1PVH | 3.540 | 21 | 0.000 | 0.000 | 0.048 | 0.000 |  |
| 1XU1 | 4.190 | 1 | 0.000 | 0.000 | 0.000 | 0.000 |  |
| 1ZHH | 3.940 | 2 | 0.000 | 0.000 | 0.000 | 0.000 |  |
| 2B4J | 3.555 | 1 | 0.000 | 0.000 | 0.000 | 0.000 |  |
| 2O3B | 4.675 | 3 | 0.000 | 0.000 | 0.338 | 0.000 |  |
| 2OOR | 2.675 | 42 | 0.000 | 0.000 | 0.000 | 0.000 |  |
| 1RV6 | 4.865 | 392 | 0.084 | 0.083 | 0.020 | 0.005 |  |
| 1JWH | 1.880 | 192 | 0.141 | 0.188 | 0.063 | 0.005 |  |
| 1OC0 | 3.430 | 333 | 0.021 | 0.039 | 0.036 | 0.015 |  |
| 2VDB | 2.755 | 99 | 0.149 | 0.020 | 0.020 | 0.020 |  |
| 3BP8 | 1.860 | 43 | 0.211 | 0.163 | 0.047 | 0.023 |  |
| 2ABZ | 4.760 | 40 | 0.025 | 0.075 | 0.026 | 0.025 |  |
| 2I9B | 5.300 | 31 | 0.130 | 0.065 | 0.130 | 0.032 |  |
| 2IDO | 6.650 | 212 | 0.120 | 0.061 | 0.005 | 0.038 |  |
| 1H9D | 7.140 | 276 | 0.022 | 0.004 | 0.018 | 0.044 |  |
| 1OFU | 3.180 | 102 | 0.000 | 0.138 | 0.000 | 0.049 |  |
| 1R6Q | 5.905 | 16 | 0.000 | 0.314 | 0.251 | 0.063 |  |
| 1FFW | 5.460 | 688 | 0.004 | 0.079 | 0.043 | 0.074 |  |
| 3CPH | 2.835 | 50 | 0.321 | 0.101 | 0.060 | 0.080 |  |
| 3SGQ | 5.615 | 115 | 0.017 | 0.140 | 0.106 | 0.105 |  |
| 1LFD | 4.310 | 105 | 0.019 | 0.249 | 0.029 | 0.106 |  |
| 1GL1 | 5.990 | 695 | 0.194 | 0.060 | 0.033 | 0.128 |  |
| 1JK9 | 4.975 | 75 | 0.603 | 0.419 | 0.067 | 0.134 |  |
| 1ZM4 | 1.715 | 347 | 0.000 | 0.154 | 0.026 | 0.151 |  |
| 1JTG | 6.805 | 369 | 0.096 | 0.145 | 0.011 | 0.155 |  |
| 2J0T | 4.810 | 36 | 0.756 | 0.168 | 0.000 | 0.168 |  |
| 1CLV | 5.515 | 1398 | 0.174 | 0.098 | 0.015 | 0.177 |  |
| 4CPA | 4.070 | 1526 | 0.112 | 0.046 | 0.015 | 0.182 |  |
| 2A9K | 4.540 | 11 | 0.000 | 0.183 | 0.000 | 0.182 |  |
| 2G77 | 5.045 | 297 | 0.054 | 0.010 | 0.000 | 0.231 |  |
| 2OUL | 5.285 | 414 | 0.337 | 0.209 | 0.057 | 0.256 |  |
| 1HCF | 5.470 | 71 | 0.071 | 0.000 | 0.000 | 0.256 |  |
| 2Z0E | 5.895 | 28 | 0.036 | 0.072 | 0.000 | 0.287 |  |
| 1WDW | 4.045 | 148 | 0.722 | 0.284 | 0.007 | 0.348 |  |
| 1FLE | 6.245 | 114 | 0.403 | 0.274 | 0.026 | 0.363 |  |
| 1JIW | 4.095 | 27 | 0.000 | 0.000 | 0.037 | 0.373 |  |
| 1OYV | 5.735 | 182 | 0.723 | 0.210 | 0.011 | 0.499 |  |
| 2A5T | 3.640 | 9 | 0.000 | 0.447 | 0.000 | 0.558 |  |
| 3D5S*^b^* | 9.720 | 105 | 0.367 | 0.183 | 0.059 | 0.613 |  |
| 2OZA*^b^* | 8.520 | 18 | 0.000 | 0.056 | 0.000 | 0.670 |  |
| 2AYO*^b^* | 6.045 | 174 | 0.208 | 0.134 | 0.052 | 0.792 |  |
| 1SYX*^b^* | 5.615 | 106 | 0.000 | 0.576 | 0.010 | 0.927 |  |

*^a^*The percentage of the near native interface areas relative to the total surface areas of the native complex; *^b^*TotalHits is the number of the hits found in the top 10000 predictions for each case.


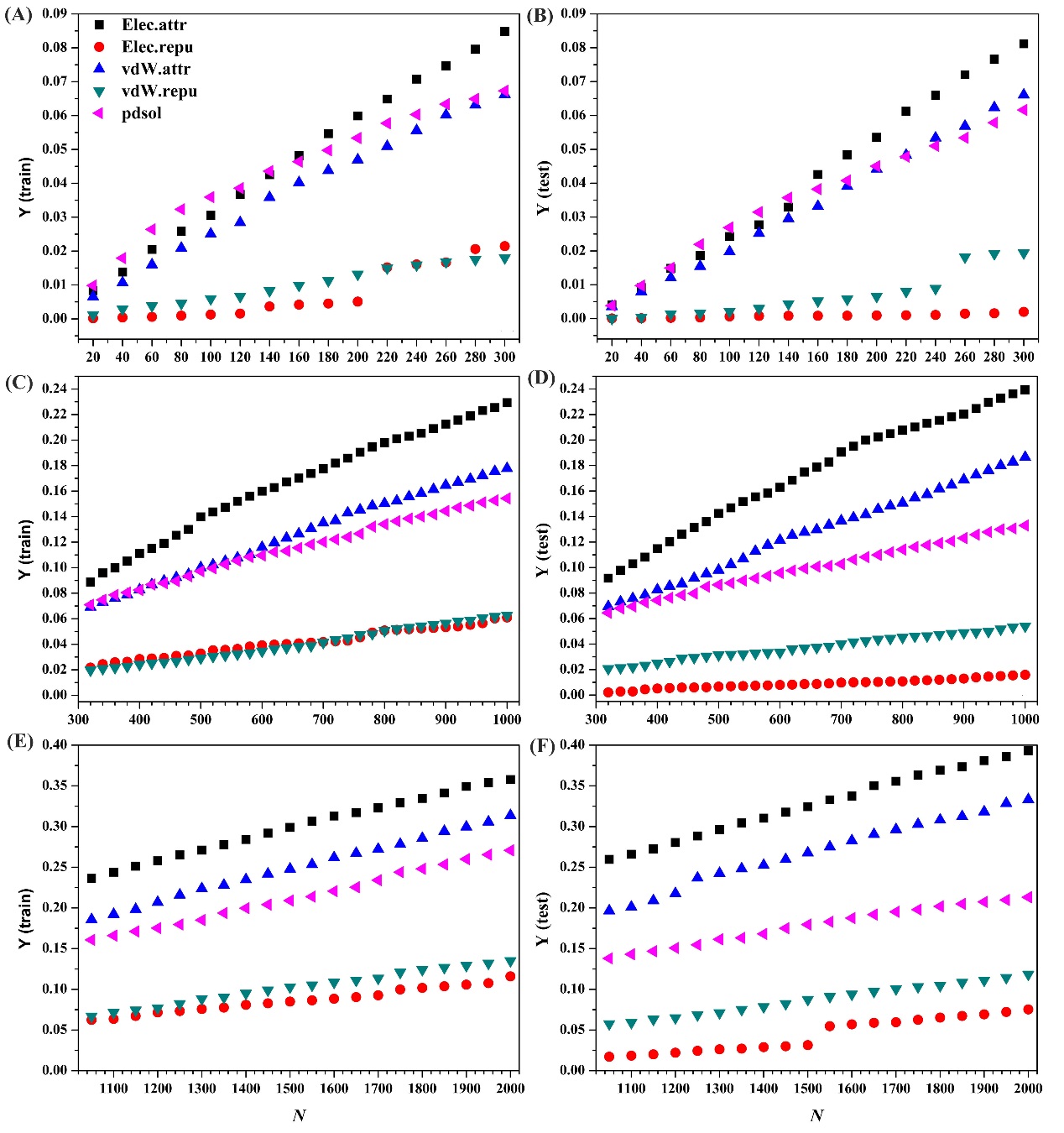


**Figure S1.** The Y curves for the five energy terms in HawkRank: (A) *N* from 20 to 300 for the training set, (B) *N* from 20 to 300 for the test set, (C) *N* from 320 to 1000 for the training set, (D) *N* from 320 to 1000 for the test set, (E) *N* from 1050 to 2000 for the training set, and (F) *N* from 1050 to 2000 for the test set.
